# Transcriptional Silencing of the Wnt-Antagonist DKK1 by Promoter Methylation Is Associated with Enhanced Wnt Signaling in Advanced Multiple Myeloma

**DOI:** 10.1371/journal.pone.0030359

**Published:** 2012-02-17

**Authors:** Kinga A. Kocemba, Richard W. J. Groen, Harmen van Andel, Marie José Kersten, Karène Mahtouk, Marcel Spaargaren, Steven T. Pals

**Affiliations:** 1 Department of Pathology, Academic Medical Center, University of Amsterdam, Amsterdam, The Netherlands; 2 Department of Hematology, Academic Medical Center, University of Amsterdam, Amsterdam, The Netherlands; Northwestern University Feinberg School of Medicine, United States of America

## Abstract

The Wnt/β-catenin pathway plays a crucial role in the pathogenesis of various human cancers. In multiple myeloma (MM), aberrant auto-and/or paracrine activation of canonical Wnt signaling promotes proliferation and dissemination, while overexpression of the Wnt inhibitor Dickkopf1 (DKK1) by MM cells contributes to osteolytic bone disease by inhibiting osteoblast differentiation. Since *DKK1* itself is a target of TCF/β-catenin mediated transcription, these findings suggest that DKK1 is part of a negative feedback loop in MM and may act as a tumor suppressor. In line with this hypothesis, we show here that *DKK1* expression is low or undetectable in a subset of patients with advanced MM as well as in MM cell lines. This absence of *DKK1* is correlated with enhanced Wnt pathway activation, evidenced by nuclear accumulation of β-catenin, which in turn can be antagonized by restoring *DKK1* expression. Analysis of the *DKK1* promoter revealed CpG island methylation in several MM cell lines as well as in MM cells from patients with advanced MM. Moreover, demethylation of the *DKK1* promoter restores *DKK1* expression, which results in inhibition of β-catenin/TCF-mediated gene transcription in MM lines. Taken together, our data identify aberrant methylation of the *DKK1* promoter as a cause of *DKK1* silencing in advanced stage MM, which may play an important role in the progression of MM by unleashing Wnt signaling.

## Introduction

Multiple myeloma (MM), one of the most common hematological malignancies in adults, is characterized by a clonal expansion of malignant plasma cells in the bone marrow, associated with suppression of normal hematopoiesis, renal failure, and osteolytic bone lesions [1], [2]. These bone lesions have been shown to be the result of uncoupled or imbalanced bone remodeling with decreased formation and increased resorption of bone tissue, due to impaired osteoblast differentiation and aberrant osteoclast activation [3]. Recent studies have identified canonical Wnt signaling as a key signal pathway in both normal bone homeostasis and in the pathogenesis of MM bone disease [4]–[6].

The canonical Wnt/β-catenin signaling pathway plays a central role in modulating the delicate balance between stemness and differentiation in several adult stem cell niches, including the intestinal crypt and the hematopoietic stem cell niche in the bone marrow [7]–[9]. *Wnt* genes encode a family of 19 secreted glycoproteins, which promiscuously interact with several Frizzled (FRZ) receptors and the low-density lipoprotein receptor-related protein 5/6 (LRP5/6). The key event in the Wnt signaling pathway is the stabilization of β-catenin. Signaling by Wnt proteins results in inhibition of glycogen synthase kinase-3β (GSK3β) activity and dissociation of the adenomatous polyposis coli (APC)/axin complex, resulting in accumulation of β-catenin, which translocates to the nucleus. Here, β-catenin interacts with T cell factor (TCF) transcription factors to drive transcription of target genes [10]. The Wnt pathway is regulated by a large number of antagonists, including the secreted frizzled-related proteins (sFRPs) and the Dickkopf (DKK) family proteins. These two classes of antagonists either act by direct binding to the Wnt ligands (the sFRPs) or by interacting with the LPR5/6 coreceptors, preventing binding of the Wnt proteins to the FRZ/LRP receptor complex (the DKKs) [11].

Recent studies indicate that the Wnt signaling plays at least two distinct roles in the pathogenesis of MM. On the one hand, studies by our own laboratory [12] as well as by the Anderson laboratory [13] have demonstrated that MMs can display aberrant activation of the canonical Wnt signaling pathway. This Wnt pathway activation presumably results from auto- and/or paracrine stimulation by Wnts, and promotes tumor proliferation and dissemination [12], [13]. On the other hand, as first shown by Tian and colleagues [6], MMs overexpress and secrete the Wnt signaling inhibitor Dickkopf-1 (DKK1), which contributes to osteolytic bone disease by inhibiting osteoblast differentiation [14]–[17]. Similar to DKK1, secretion of the Wnt inhibitor sFRP2 by MM cells may also promote myeloma bone disease [5]. Since both DKK1 and sFRP2 are established targets of TCF/β-catenin-mediated transcription [18]–[20], these findings suggests the presence of a negative feedback loop in MM in which DKK1 and sFRP2 act as potential tumor suppressors. In line with this hypothesis, we show here that DKK1 expression is often low or undetectable in advanced myeloma and is absent in MM cell lines, which are generally derived from advanced extramedullary myeloma. This silencing of DKK1 is caused by methylation of the *DKK1* promoter and unleashes β-catenin/TCF mediated transcription.

## Materials and Methods

### Ethics Statements

The study involving human biopsy samples was conducted in accordance with the Declaration of Helsinki and approved by the local ethics committee of The University of Amsterdam, AIEC (Algemene Instellingsgebonden Ethische Commissie). Patients gave written informed consent for the sample collection.

### Case selection and classification

A panel of BM biopsy specimen from 41 MM and 7 MGUS patients, obtained at clinical diagnosis, was selected from the files of the Department of Pathology, Academic Medical Center, University of Amsterdam, Amsterdam, The Netherlands. All patients were staged according to the Salmon–Durie system. For statistical analysis patients at stage I and II disease were grouped together (n = 16) and classified as early MM, whereas patients with stage III disease (n = 25) were classified as having advanced MM.

### Microarray analysis

For the analysis of expression of Wnt family members in MM patients, expression data publically available and deposited in the NIH Gene Expression Omnibus (GEO National Center for Biotechnology Information [NCBI], http://www.ncbi.nlm.nih.gov/geo/ were used. These concerned the U133 Plus2.0 affymetrix oligonucleotide microarray data from 559 newly diagnosed MM patients included in total therapy 2/3 (TT2, TT3), provided by the University of Arkansas for Medical Sciences, GSE2658 [21].

### Immunohistochemistry/Immunocytochemistry

Immunohistochemical staining was performed on formalin-fixed, plastic-embedded bone marrow sections as described previously [22]. Sections were deplastified in acetone, after which endogenous peroxidase was blocked with a 0.3% solution of H_2_O_2_ in methanol and followed by antigen retrieval for 10 minutes in TRIS/EDTA buffer (respectively 10 mM/1 mM) pH 9.0 at 100°C. After blocking with serum free blocker (DAKO, Carpinteria, CA), the slides were either incubated for one hour at room temperature with anti-CD138 (IQP-153 IQ Products, Groningen, The Netherlands) or overnight at 4°C with anti-DKK1 (Abcam, Cambridge, MA), or anti-β-catenin (clone 14 BD Biosciences, Erembodegem, Belgium). For CD138 and β-catenin, binding of the antibody was visualized using the PowerVision plus detection system (Immunovision Technologies, Duiven, The Netherlands) and 3, 3-diaminobenzidine (Sigma-Aldrich, St Louis, MO). Whereas binding of the DKK1-antibody was visualized with a biotinylated rabbit anti-goat antibody, followed by horseradish peroxidase (HRP)-conjugated streptavidin (DAKO) and DAB^+^ (DAKO). The sections were counterstained with hematoxylin (Merck, Darmstadt, Germany), washed and subsequently dehydrated through graded alcohol, cleared in xylene, and coverslipped. Immunocytochemical staining was performed on formalin-fixed cells. Briefly, slides were incubated for 30 minutes with PBS 0.3% Triton X-100 followed by blocking with serum free blocker (DAKO, Carpinteria, CA), the slides were then incubated overnight at 4°C with anti-DKK1 (goat polyclonal R@D). Binding of the DKK1-antibody was visualized with a biotinylated rabbit anti-goat antibody, followed by horseradish peroxidase (HRP)-conjugated streptavidin (DAKO) and 3-amino-9-ethyl carbazole (AEC).

The biopsies were analyzed for DKK1 and β-catenin expression by two independent observers (RWJG and STP). DKK1 expression was scored in three semi-quantitative categories, *i.e*., low (0–25%), intermediate (25–75%) and high (75–100%), with the percentages indicating the number of DKK1 positive MM plasma cells. For β-catenin expression the intensity of nuclear staining was scored as negative/low, intermediate or high. For statistical analysis, the cases scored as negative/low and intermediate were grouped together as low β-catenin.

### Methylation analysis

Genomic DNA was extracted and purified from MM bone marrow suspensions and cell lines using DNAzol (Invitrogen Life technologies, Breda, The Netherlands) according to the manufacturer's protocol. The extracted DNA was modified by treatment with sodium bisulfite using the EpiTect Bisulfite Kit (Qiagen, Hilden, Germany). The methylation status of the *DKK1* gene was assessed using both methylation specific PCR (MSP) and bisulfate sequencing, as described by Aquilera et al. [23].

### Cell culture and demethylation treatment

MM cell lines L363, UM-1, OPM-1, RPMI 8226, and PC-3 were cultured in RPMI medium 1640 (Invitrogen Life technologies) containing 10% clone I serum (HyClone), 100 units/ml penicillin, and 100 µg/ml streptomycin. XG-1 and LME-1 cell lines were cultured in IMDM medium (Invitrogen Life technologies) supplemented with transferrin (20 µg/ml) and β-mercaptoethanol (50 µM). For XG-1, medium was additionally supplemented with IL-6 (500 pg/m). 293T cells were cultured in DMEM medium containing 10% clone I serum (HyClone), 100 units/ml penicillin, and 100 µg/ml streptomycin. The PC-3 cell line was kindly provided by Christopher Hall. L-cells and Wnt3a-producing L-cells (L-306.72 Mouse L fibroblasts, stably transfected with Wnt3a) were cultured in DMEM medium (Invitrogen Life technologies) containing 10% clone I serum (HyClone), 100 units/ml penicillin, and 100 µg/ml streptomycin. Conditioned medium was harvested from 95% confluent flasks every 72 h and stored at 4°C. 5-aza-2′-deoxycytidine (5-aza-CdR Sigma-Aldrich) treatment was performed for 72 h at a final concentration of 5 µM, after which they were harvested and lysed in Trizol (Invitrogen Life technologies).

### RT-PCR

Total RNA was isolated using Trizol according to the manufacturer's protocol (Invitrogen Life technologies). The RNA was further purified using iso-propanol precipitation and was concentrated using the RNeasy MinElute Cleanup kit (Qiagen). The quantity of total RNA was measured using a NanoDrop ND-1000 Spectrophotometer (NanoDrop Technologies, Wilmington, DE). 5 µg of total RNA was used for cDNA synthesis as described previously. The PCR mixture contained: 100 ng of cDNA, 1× PCR Rxn buffer (Invitrogen Life technologies), 0.2 mmol/L dNTP, 2 mmol/L MgCl_2_, 0.2 µmol/L of each primer, and 1 U platinum Taq polymerase (Invitrogen Life technologies). PCR conditions were: denaturing at 95°C for 5 minutes, followed by 30 cycles of 30 s at 95°C, 30 s at 65°C (DKK1), 55°C (β-actin) and 30 s at 72°C. The reaction was completed for 10 minutes at 72°C. Primers used were: DKK1 forward (5′–GATCATAGCACCTTGGATGGG–3′) DKK1 reverse (5′–CAGTCTGATGACCGG–3′) β-actin forward (5′–GGATGCAGAAGGAGATCACTG–3′) β-actin reverse (5′–TCCACACGGAGTACTTG–3′). All primers were manufactured by Sigma-Aldrich (Haverhill, UK).

### Western blot analysis

Conditioned medium was directly lysed in sample buffer, separated by SDS/10% PAGE, and blotted. Primary goat anti-DKK1 antibody (R@D) was detected by a horseradish peroxidase-conjugated swine anti-goat antibody, followed by detection using Lumi-Light PLUS western blotting substrate (Roche). Protein was harvested from MM cell lines, fractionated using a nuclear/cytosol fractionation kit (BioVision), separated by 10% SDS-polyacrylamide gel electrophoresis and subsequently blotted. The following antibodies were used: β-catenin (clone 14 BD Biosciences, Erembodegem, Belgium), β-tubulin (SantaCruz Biotechnologies), and histone H2B (Imgenex). Primary antibodies were detected by HRP-conjugated secondary antibodies, followed by detection using Lumi-Light PLUS western blotting substrate (Roche).

### Retroviral vector production and transduction of MM cell lines

The LZRS-pBMN-DKK1-IRES-eGFP retroviral vector encoding *DKK1* and *eGFP* gene was generated by inserting the *DKK1* gene from pcDNA3.1 into the BamHI and XhoI sites of LZRS-pBMN-IRES-eGFP vector (S-001-AB provided by Dr. G. Nolan). The amphotropic Phoenix packaging cell line was transfected either with LZRS-pBMN-IRES-eGFP or LZRS-pBMN-DKK1-IRES-eGFP by the use of Fugene (Roche). The selection of transfected cells was performed with puromycine. Virus was harvested from the packaging cell lines when 95% of the cells were eGFP-positive as analyzed by fluorescence activated cell sorting (FACS). Transduction of MM cell lines was done by 16 h of incubation with virus supernatant in the presence of 10 µg/ml retronectin (Takara Biomedicals, Shiga, Japan). 2 days after the transduction eGFP positive cells were analyzed and sorted by FACS. The transduced MM cells were expanded and examined regularly for eGFP and DKK1 expression. The expression of DKK1 protein was determined in conditioned medium by western blot analysis.

### Luciferase assay

MM cells (10 mln) were transfected with TOPFLASH reporter construct (5 ug) and pRL-TK (1 ug) in 500 ul serum-free medium. Treatment with Wnt3a conditioned medium or L-cell conditioned medium was initiated 24 h upon transfection and subsequently maintained for 24 h. Next, MM cells were lysed in 100 ul passive lysis buffer (Promega) and firefly luciferase activity and *Renilla* luciferase activity were measured using the dual luciferase assay kit (Promega) following the manufacturers instruction. *Renilla* luciferase activity served as an internal control for transfection efficiency.

### Statistical analysis

The chi-square (χ^2^) test was used to test the correlation between the DKK1 expression and the stage of disease, the relation between expression of nuclear β-catenin and the stage of disease and the correlation between expression of DKK1 and nuclear β-catenin.

## Results

### Wnt signaling activation is associated with advanced stage MM

Previous studies have demonstrated that the malignant plasma cells in MM can display Wnt pathway activation. Since no mutations in Wnt pathway components were detected, this activation is presumably caused by auto- and/or paracrine stimulation by Wnt ligands secreted in the bone marrow microenvironment. [12], [13]. In support of a ligand-dependent activation scenario, analysis of gene expression profiling data of the malignant plasma cells of 345 MM patients (http://www.ncbi.nlm.nih.gov/geo/ accession number GSE2658) revealed frequent (co-)expression of various Frizzleds (e.g. Fzd 1, 3, 6, 7 and 8) and the co-receptor LRP6 (LRP5 could not be studied because of defective probe sets), as well as various Wnts (e.g. 4, 5A, 5B 6, 10A and 16). Moreover, 51% of the primary MMs co-express the co-receptor LRP6 with at least one of the Frizzled genes and one of the Wnt genes (Table S1). Hence, in the majority of MM patients the malignant plasma cells appear to be well equipped to evoke autocrine activation of the Wnt pathway. Furthermore, Wnt ligands are produced by bone marrow stromal cells [12], [24], [25], which may cause paracrine Wnt pathway activation.

**Figure 1:**
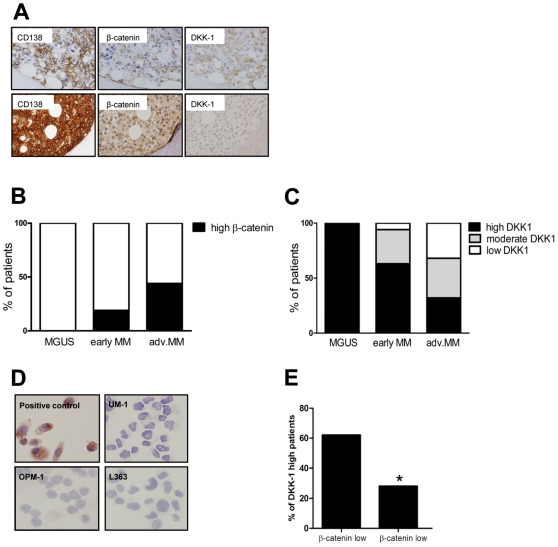
The relation between the Wnt pathway activation and the loss of DKK1 expression during MM progression. (A) Representative pictures of immunohistochemical staining of a multiple myeloma patient displaying either high DKK-1 and low β-catenin expression (upper panel) or with DKK-1 loss and increased nuclear β-catenin localization (lower panel). Immunohistochemical stainings are shown for CD138 (left column), β-catenin (middle column) and DKK1 (right column). (B) Nuclear β-catenin expression in relation to multiple myeloma progression (n = 48, p<0.05). (C) DKK-1 expression in relation to multiple myeloma progression (p<0.05). (D) Representative pictures of immunocytochemical staining of multiple myeloma cell lines with goat polyclonal anti-DKK1 antibody (magnification: 400×). Prostate cancer cell line (PC-3) was used as positive control (PC) for the DKK-1 staining. (E) Relation between the loss of DKK-1 expression and nuclear localization of β-catenin (p>0.05). A significant correlation (p<0.05) between expression of nuclear β-catenin and DKK-1 was observed in the two extreme groups identified based on β-catenin expression. * indicates p value<0.05.

To explore whether MM cells localized within the bone marrow microenvironment indeed display evidence of active Wnt signaling, we analyzed a panel of MM bone marrow biopsies for nuclear expression of β-catenin, a key feature of active canonical Wnt signaling [22], [26]. The panel consisted of 41 MM and 7 MGUS biopsies patients all biopsies were obtained at first diagnosis. Immunohistochemical studies revealed strong β-catenin staining in the malignant plasma cells of 34% of the MM patients (Figure 1 A–B). Interestingly, as shown in Figure 1B, β-catenin expression was significantly correlated with disease stage: nuclear β-catenin was present in none of the MGUS patients, 19% of the stage I/II patients, and 44% of the patients with advanced MM (p<0.05). These findings imply the presence of active Wnt signaling in BM-cocalized MM cells and show that this is associated with advanced stage MM.

### Absence of DKK1 expression unleashes Wnt pathway activation in MM

Since stimulation by (autocrine and/or paracrine) Wnt ligands appears to underly Wnt pathway activation in MM, loss of secreted Wnt pathway antagonists like DKKs and sFRPs could have a major impact on the pathogenesis of MM. In particular DKK1, which binds to LRP5/6 thereby preventing activation of the pathway by Wnt ligands, might act as a potent negative regulator as it has been shown to be overexpressed by MM cells [18]–[20]. To gain insight into the expression of the DKK1 protein in primary MM samples, we studied *DKK1* expression in the above described panel of BM biopsies. DKK1 was detected in most (81%) of the biopsies. However, in a significant proportion expression was either restricted to a subfraction of the malignant plasma cells or was entirely lost (Figure 1 A, C). In contrast to expression of β-catenin, DKK1 expression was negatively correlated with disease stage: whereas all MGUS patients and the majority of early (stage I/II) MM patients demonstrated high DKK1 expression, DKK1 was either partially or completely absent in 68% of the patients with advanced MM (p<0.05) (Figure 1C). Furthermore, DKK1 protein expression was not detected in any of the studied MM cell lines (Figure 1D). Direct comparison between the level of DKK1 and nuclear β-catenin expression in individual patients indeed revealed a significant inverse correlation between these parameters (p<0.05) (Figure 1E). Our results demonstrate a correlation between DKK1 expression and MM stage with a low or undetectable levels of *DKK1* in a subset of patients with advanced stage MM (p<0.05), and an inverse relation to nuclear β-catenin expression.

**Figure 2:**
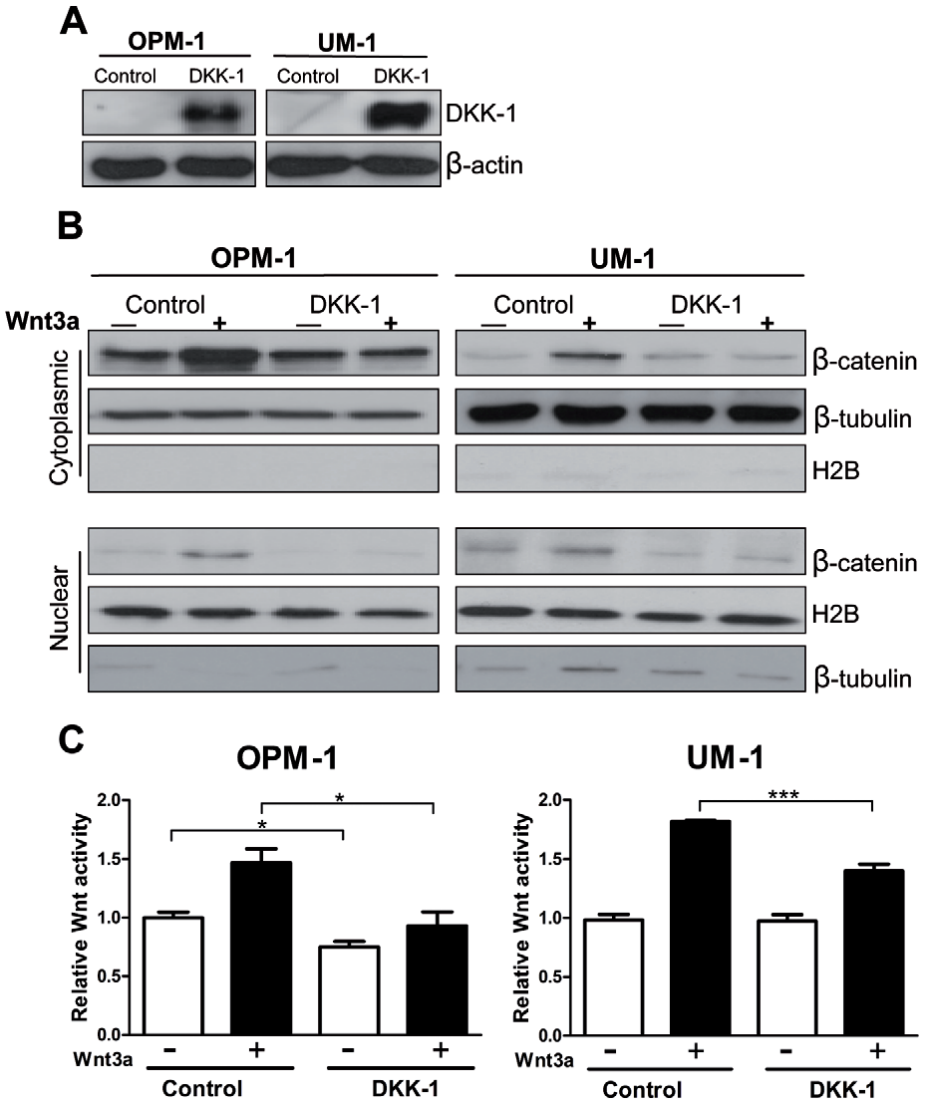
DKK1 expression represses Wnt pathway activation in MM. (A) MM cell lines OPM-1 and UM-1 were transduced with either the LZRS-pBMN-IRES-eGFP (control) or the LZRS-pBMN-DKK1-IRES-eGFP (DKK1) virus. Conditioned medium of sorted, transduced cells was harvested and immunoblotted using a goat polyclonal antibody against DKK1. Representative immunoblot confirms the expression of DKK1 in the conditioned medium of LZRS-pBMN-DKK1-IRES-eGFP transduced cells. β-actin is shown as internal control for equal cell number. (B) Cytoplasmic and nuclear proteins were prepared from the LZRS-pBMN-IRES-eGFP (control) or the LZRS-pBMN-DKK1-IRES-eGFP (DKK1) MM cells, stimulated for 24h with Wnt3a conditioned medium (+). As a control, L-cells conditioned medium was applied (−).To assess β-catenin accumulation, nuclear and cytoplasmic cells lysate was immunoblotted by using a monoclonal anti-β-catenin antibody. The bottom part of the blot was stained with β-tubulin and Histone H2B as controls for cytoplasmic and nuclear proteins, respectively. (C) LZRS-pBMN-IRES-eGFP (control) or the LZRS-pBMN-DKK1-IRES-eGFP (DKK1) cells were transfected with TOPFLASH reporter and renilla contruct. 24 hours upon transfection cells were treated with L-cells conditioned medium (−) or Wnt3a conditioned medium (+).The relative light units value of LZRS-pBMN-IRES-eGFP cells treated with L-cells conditioned medium was normalized to 1. The mean ± SD of representative experiment performed in triplicate is shown. * indicates p value<0.05 *** indicates p value<0.001. by student's t test.

The above findings suggest that silencing of *DKK1* may unleash activation of the canonical Wnt pathway by Wnt ligands, leading to increased levels of nuclear β-catenin in MM cells of patients with advanced disease. To explore whether DKK1 expression by MM cells can indeed control the response of MM cells to ligand-induced Wnt pathway activation, we restored the DKK1 expression in the MM cell lines OPM-1 and UM-1 by retroviral transduction (Figure 2A). OPM-1 and UM-1 lack mutations in Wnt pathway components and co-express Frizzleds, the co-receptor LRP6, and Wnts, and have been reported to display constitutively active β-catenin/TCF-dependent transcription [12], [27], [28]. As shown in Figure 2B, introduction of *DKK1* expression in OPM-1 and UM-1 cells leads to reduced Wnt3a induced and baseline β-catenin levels. Furthermore, it results in a significant inhibition of ligand-induced TCF-mediated transcription (Figure 2C). These findings suggest that *DKK1* expression by MM cells could indeed restrain the response of these cells to Wnt ligands expressed in the BM microenvironment.

### *DKK1* promoter hypermethylation in MM cell lines and primary tumors

**Figure 3:**
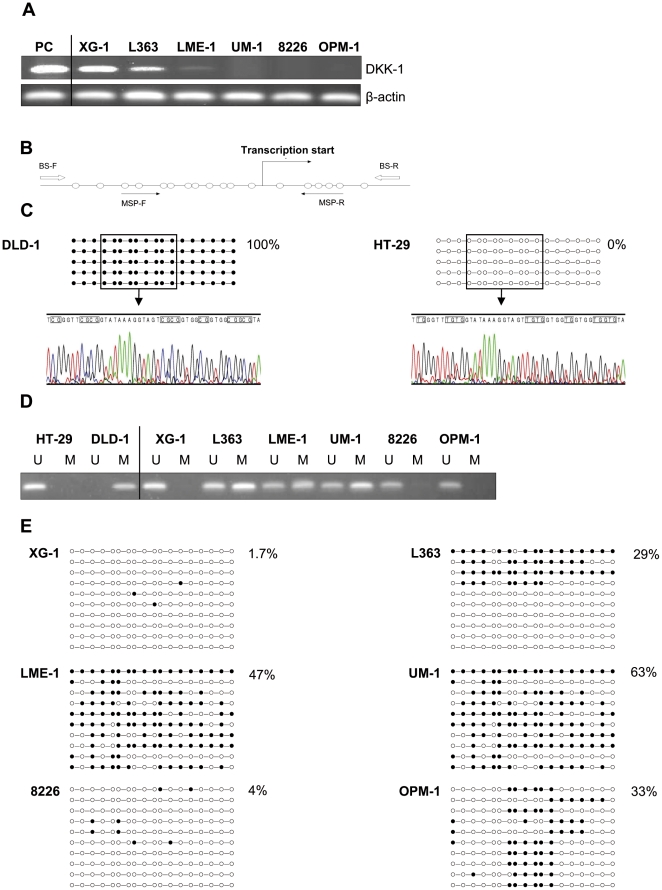
*DKK1* promoter methylation in MM cell lines. (A) Total RNA was isolated and RT–PCR analyses were performed with the specific primers indicated. Complementary DNA from prostate cancer cell line (PC-3) was used as positive control (PC) for DKK1 expression. The β-actin expression was used as a loading control. (B) Schematic representation of the promoter area analyzed for *DKK1*, containing a CpG island. White arrows indicate the positions of primers used for bisulfite sequencing, and black arrows indicate the positions of primers used for methylation specific PCR. Each of the CpG dinucleotides is presented as open circle. (C) *Upper panel*. Representation of bisulfite genomic sequencing results of 5 clones of the *DKK1* promoter region in HT-29 and DLD-1 colon cell lines used as unmethylated (U_DNA) and methylated (M_DNA) control, respectively. The amplified 326 bp product corresponds to the *DKK1* promoter region from −193 to +122. In total, 18 CpG dinucleotides (CpGs) within the CpG island were analyzed and are represented as open and closed circles, which indicate unmethylated and methylated CpG sites, respectively. *Lower panel*. Electropherograms of bisulfite modified DNA from *DKK1* CpG island in HT-29 (U_DNA) and DLD-1 (M_DNA) cells. (D) Methylation specific PCR of the CpG island of the *DKK1* promoter region in MM cell lines. DNA bands in lanes labeled with U indicate PCR products amplified with primers recognizing unmethylated promoter sequences, whereas DNA bands in lanes labeled with M represent amplified products with primers designed for the methylated template. (E) Bisulfite sequencing analysis of the the *DKK1* promoter region in multiple myeloma cell lines, open circles indicating unmethylated CpG sites, and closed circles representing methylated CpG sites. Percentages indicate the fraction of methylated CpG dinucleotides of the total CpG sites analyzed.

To further analyze the absence of *DKK1* expression in MM, we examined MM cell lines for *DKK1* mRNA expression. In MM cell lines, which all lack detectable levels of DKK1 protein (Figure 1D and Figure S1), the *DKK1* transcript was either undetectable (UM-1, RPMI 8226 and OPM-1), or weakly expressed (XG-1, L363 and LME-1) in comparison to the *DKK1* expression in the (positive control) prostate cancer cell line PC-3 (Figure 3A) [29].

Since it has been reported that *DKK1* is a target for epigenetic inactivation by CpG island promoter hypermethylation in several forms of cancer [23], [30]–[33], we hypothesized that epigenetic silencing could also be responsible for the lack of DKK1 in primary MM cells and cell lines. Hence, we studied the *DKK1* promoter region, including the CpG island encompassing the transcriptional start site for methylation using a methylation specific PCR (MSP) and a bisulphate-sequencing PCR (BSP) (Figure 3B). DNA isolated from the colon cancer cell lines HT-29 and DLD-1, previously reported to be unmethylated and methylated, respectively [23], was used to validate our experimental set-up (Figure 3C).

Combined MSP and BSP analysis revealed that four of the 6 MM cell lines tested, *i.e*., L363, LME-1, UM-1, and OPM-1, showed hypermethylation of the *DKK1* promoter, while XG-1 and RMPI8226 were unmethylated (Figure 3 D–E). Notably, the BSP analysis of OPM-1 revealed a methylation pattern that was not detected by the MSP primers, thus showing the necessity of using both techniques to adequately perform methylation studies. Interestingly, in the L363 cell line four out of ten sequenced clones displayed extensive CpG methylation, suggesting that either a subset of the cells show methylation or that there is partial monoallelic methylation within individual cells. The lack of DKK1 expression despite the absence of promoter methylation in RPMI8226 cells indicates either the mere lack of transcriptional activation or an alternative mechanism of *DKK1* silencing.

**Figure 4:**
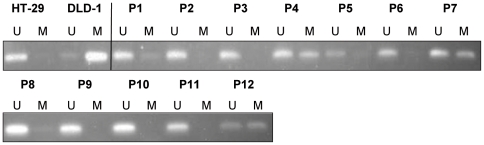
Analysis of *DKK1* promoter methylation in MM bone marrow samples. Methylation specific PCR of the CpG island of the *DKK1* promoter region in the bone marrow samples of twelve patients with multiple myeloma (P1–P12), HT-29 and DLD-1 colon cell lines were used as unmethylated (U_DNA) and methylated (M_DNA) control respectively. DNA bands in lanes labeled with U and M indicate PCR products amplified with primers recognizing unmethylated and methylated promoter sequences respectively.

Since *DKK1* expression appears to be decreased in MM cells isolated from patients with advanced stage MM, we next investigated the *DKK1* promoter associated CpG island in the bone marrow mononuclear cells (BM-MNC) of patients with MM and compared this to BM-MNC from healthy donors. All 12 patients had stage III disease. The *DKK1* promoter was hypermethylated in 33% of the primary MM specimens studied, as determined by BSP (Figure S2) and MSP analysis (Figure 4). In accordance with a previous study [32], none of the healthy donor BM samples tested displayed methylation of the *DKK1* promoter (data not shown).

### Promoter methylation silences *DKK1* expression

**Figure 5:**
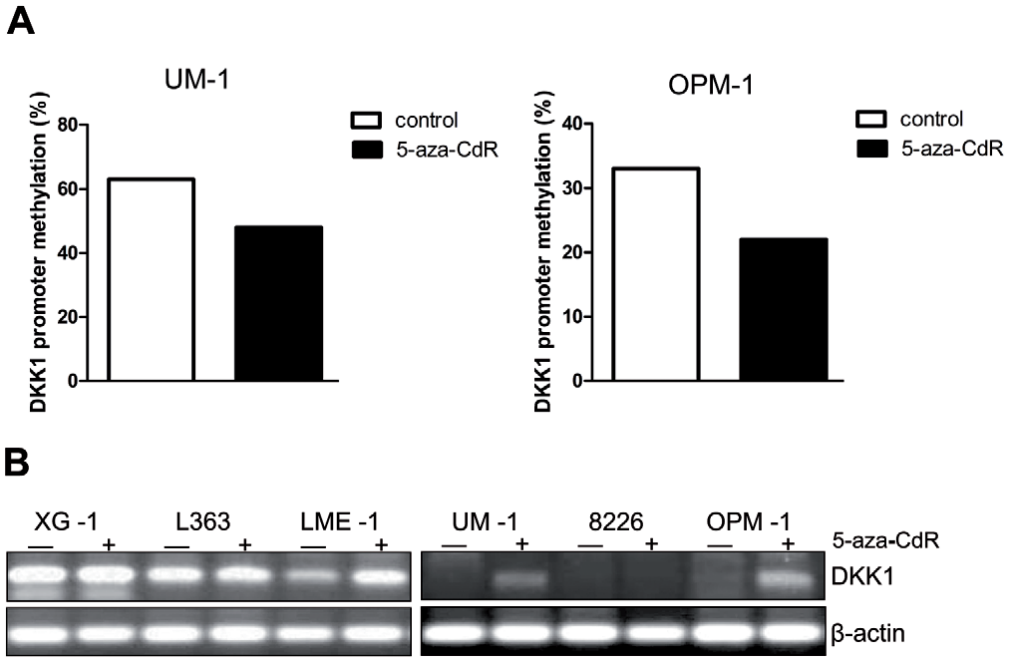
Restoration of *DKK1* expression in MM cell lines by 5-aza-2-deoxycytidine treatment. (A) *DKK-1* promoter methylation analyzed by bisulfite genomic sequencing of 10 clones, on DNA isolated from 5-aza-2-deoxycytidine treated (5-aza-CdR) and untreated (PBS) MM cell lines UM-1 and OPM-1. Frequency of methylation was calculated by dividing the number of methylated CpG sites by the total number of analyzed CpG sites. (B) Reverse transcriptase-PCR analysis for DKK1 gene expression in multiple myeloma cell lines in the absence and presence of the demethylating agent 5-aza-2-deoxycytidine. β-actin expression is shown as an input control.

To assess whether *DKK1* promoter methylation indeed plays a causative role in the transcriptional silencing of *DKK1* in MM, we investigated whether *DKK1* expression could be restored or enhanced by treatment with the demethylating agent 5-aza-2-deoxycytidine. Analysis by BSP of the genomic DNA isolated from OPM-1 and UM-1 cells, which combine a lack of DKK1 expression with hypermethylation of its promoter (Figures 1D and Figure 3), confirmed that treatment results in a decrease in methylation of the *DKK1* promoter (Figure 5A). Importantly, treatment of these cell lines results in restoration of *DKK1* expression (Figure 5B). In addition, in LME-1, a cell line with a hypermethylated *DKK1* promoter and low *DKK1* expression, *DKK1* expression was increased upon 5-aza-2-deoxycytidine treatment. As expected, treatment of XG-1 and RPMI8226, the two cell lines that lack methylation of the *DKK1* promoter, did not affect the expression (Figure 5B). Taken together, these data establish a direct role for CpG island methylation in epigenetic silencing of *DKK1* expression in MM.

## Discussion

We have previously demonstrated that the Wnt pathway, which is essential for normal T and B cell development [8], [34] and plays a key role in the development of several types of cancer [35], [36], is aberrantly activated in MM and can promote MM tumor growth [12]. Subsequent studies have confirmed the oncogenic potential of the Wnt pathway in MM by demonstrating that targeting the Wnt pathway by drugs or siRNAs leads to inhibition of MM growth [12], [13], [37]–[39]. In addition, by knocking down β-catenin, Dutta-Simmons et al. revealed new potential Wnt/β-catenin transcriptional targets involved in various aspects of cell-cycle progression, such as CDC25 A/B, cyclins, cyclin-dependent kinases, and AurKA/B. Among the genes significantly downregulated upon β-catenin knock down, a significant proportion had LEF/TCF4 (GCTTTGT/A) binding sites in their promoters, identifying them as putative direct Wnt target genes [13].

Although the exact cause(s) of aberrant Wnt pathway activation in MM has not yet been established, the absence of detectable Wnt pathway mutations [12] as well as the (over)expression of Wnt ligands in the BM microenvironment by both stromal cells and by the MM cells themselves [12], [28], (and table S1), suggests a key role for autocrine and/or paracrine stimulation. As a consequence, loss of secreted Wnt pathway antagonists like DKKs and sFRPs could have a major impact on the pathogenesis of MM. Indeed, we observed that whereas the DKK1 protein is strongly expressed in most primary MMs, the expression of this Wnt antagonist is down-regulated or even completely absent in a subgroup of advanced (stage III) MMs (Figure 1A, C). In addition, the DKK1 protein was undetectable in MM cell lines, which represent the ultimate, microenvironment independent, phase of MM tumor progression and are almost invariably derived from extramedullary MMs (Figure 1D and Figure S1).

Interestingly, low or undetectable of DKK1 protein expression in BM samples of MM patients was correlated with an increased nuclear expression of β-catenin, a hallmark of canonical Wnt signaling (Figure 1E). DKK1 is a major Wnt pathway antagonist which acts by interfering with the binding of Wnt ligands to the LRP5/6 coreceptor [40]. Importantly, the *DKK1* gene itself is a direct target of β-catenin/TCF-mediated transcription [18]–[20], and DKK1 has been implicated in the feed-back regulation of Wnt signaling in several biological systems [41]–[43]. Consistent with a tumor suppressor function, *DKK1* silencing during tumor progression has been reported in several types of cancer [23], [30]–[33]. Our observation, that DKK1 levels can be low or undetectable in advanced MM and that restoration of its expression inhibits β-catenin/TCF transcriptional activity (Figure 2C), suggests that silencing of *DKK1* may contribute to activation of the canonical Wnt pathway during MM progression.

Like loss of function mutations, aberrant methylation of the promoter of tumor suppressor genes can provide a selective advantage to neoplastic cells [44]–[48]. We identified *DKK1* promoter hypermethylation as a mechanism underlying the absence of DKK1 expression in MM (Figure 3, 4, 5). In four of the 6 MM cell lines tested, *i.e.*, L363, LME-1, UM-1, and OPM-1, we showed hypermethylation of the *DKK1* promoter (Figure 3). The CpG island analyzed encompasses the first exon of the *DKK1* gene, which encodes the transcriptional and translational start sites as well as a significant part of the region upstream of the coding sequence, an organization characteristic of genes targeted by epigenetic silencing [46]. Indeed, this CpG island has previously been implicated in DKK1 silencing in several types of cancer, including colorectal cancer, gastric cancer, breast cancer, medulloblastoma and leukemia [23], [30]–[33]. Importantly, the promoter methylation was reduced and *DKK1* expression was either restored and/or markedly increased by the DNA demethylating agent 5-aza-2-deoxycytidine (Figure 5A–B), confirming that the observed aberrant methylation indeed was instrumental in the silencing of *DKK1* expression (Figure 5B). Interestingly, 5-azacytidine has been reported to have significant cytotoxic activity against MM cell lines as well as patient-derived malignant plasma cells, but not against peripheral blood mononuclear cells [49]. Of the cell lines used in our study, OPM-1 and UM-1 display a very high sensitivity to 5-azacytidine and treatment of these cells with this compound result not only in promoter demethylation but also in rapid and extensive cell death, which explains the rather modest induction of DKK1 expression. Indeed, in the LME-1 cell line, which shows a lower sensitivity to 5-azacytidine-induced cell death, treatment results in a much stronger upregulation of DKK1 mRNA expression. In primary MM, we also observed dense methylation of the DKK1-associated CpG island (Figure 4 and Figure S2). Since methylation of the *DKK1* promoter was not observed in normal bone marrow samples [32], this methylation can be considered aberrant and disease-related. In addition to silencing of *DKK1*, silencing of other Wnt antagonists could also contribute to the enhanced Wnt signaling in advanced MM. Consistent with this notion, Chim *et al.*, have reported that constitutive Wnt signaling in MM cell lines is associated with methylation dependent silencing of several Wnt inhibitors, including the sFRP1, 2, 4 and 5. Methylation of at least one of these soluble Wnt inhibitors was observed in most primary MM bone marrow samples [50].

Our finding that the *DKK1* promoter is methylated and Wnt pathway is hyperactivated in advanced multiple myeloma, strongly suggests the presence of autocrine Wnt signaling in malignant plasma cells. In accordance with this hypothesis we observed inhibition of nuclear β-catenin levels and of Wnt reporter activity upon restoration of DKK1 in MM cells (Figure 2 A–C). Importantly, MM cell lines used in this experiment have dense methylation of the *DKK1* promoter around the transcription start region and lack detectable DKK1 transcript (Figure 1D, Figure 3A and Figure S1). Taken together, these data suggest that activation of Wnt signaling in these cell lines is the consequence of *DKK1* silencing and could reflect the progression-dependent Wnt pathway activation in patients with advanced MM.

Our current study, in conjunction with work of others, points to a multi-facetted role of DKK1 in the pathogenesis of MM. Studies by Shaughnessy and collegues have previously also reported that DKK1 is strongly expressed by the malignant plasma cells of most MM patients [6]. It was shown that secretion of the DKK1 can contribute to MM bone disease by inhibiting Wnt signaling in osteoblasts, thereby interfering with their differentiation [14]–[17]. Furthermore, in line with our current findings, which suggests that DKK1 may act as a feed-back tumor suppressor, these authors also reported loss of DKK1 protein expression in a subgroup of patients with advanced MM [6]. In addition to causing bone disease, inhibition of osteoblast differentiation by DKK1 may also promote MM growth, since mature osteoblasts can suppress myeloma growth, whereas immature osteoblasts express high levels of IL-6, a central growth and survival factor for myeloma plasma cells [51]. Furthermore, DKK1 enhances the expression of receptor activator of NF-kappa B ligand (RANKL) and downregulates the expression of osteoprotegerin (OPG) in immature osteoblast [17]. The resulting increased RANKL/OPG ratio leads to osteoclast activation promoting osteolytic bone disease. Osteoclasts may also support the growth of myeloma cells through secretion of IL-6 and osteopontin, and by adhesive interactions, stimulating the proliferation of malignant plasma cells [52]. Thus, like several other soluble factors expressed by MM cells, for example vascular endothelial growth factor (VEGF) and hepatocyte growth factor (HGF) [53]–[55], DKK1 can both, exercise paracrine effects on the BM microenvironment, and affect the MM cells in an autocrine fashion the net effect could be either enhanced or reduced tumor growth.

Consistent with this hypothesis, by employing a MM SCID/rab mouse model, Yaccoby et al. showed that treatment with anti-human DKK1-neutralizing antibody stimulates osteoblast activity, reduces osteoclastogenesis, and promotes bone formation in myelomatous and nonmyelomatous bones [56]. MM burden was also reduced, but notably, not in all mice bearing human myeloma cells [57]. Similar results were also obtained in a SCID/hu mouse model by Fulciniti et al [56], [57]. Together, these studies suggest that MM bone disease and tumor growth are interdependent, at least at the intramedullary stage, and that increased bone formation as a consequence of neutralization of DKK1, may also control MM growth [56], [57].. However, in a 5T2MM murine myeloma model treatment with the anti-DKK1 antibody BHQ880 also caused a reduction of osteolytic bone lesions but did not have any effect on tumor burden [58]. Give our current finding that DKK1 inhibits autocrine canonical Wnt signaling in MM cells, inhibition of DKK1 could hyperactivate the Wnt pathway and thereby promote tumor growth, especially at extramedullary sites. Indeed, stimulation of the Wnt signaling pathway in a 5TGM1 mouse myeloma model significantly increased subcutaneous tumor growth [59]. In patients, extramedullary growth is associated with aggressive disease, occurring subsequent to the osteolytic bone disease, often resulting in plasma cell leukemia. Importantly, in a human-mouse xenograft MM model, Dutta-Simmons et al. demonstrated that the Wnt pathway not only controls the proliferation of MM plasma cells but also their metastatic potential [13]. Taken together, these studies suggest a scenario in which DKK1 has a dual, stage depend, role: whereas high DKK1 expression in early MM contributes to a tumor permissive micronenviroment within the BM, advanced MMs that have acquired BM independence may benefit from DKK1 loss, which enhanced Wnt signaling and thereby promotes MM growth and dissemination. Although blocking DKK1 inhibits osteolytic bone disease *in vivo*, targeting DKK1 in MM patients could enhance Wnt pathway activity in MM plasma cells, which might increase the metastatic potential and extramedullary growth of the tumor.

In conclusion, our study establishes for the first time a relation between low or absence of DKK1 expression and the presence Wnt pathway activation during MM progression. Moreover, we demonstrate the presence of a functional ligand-dependent Wnt signaling in MM cells and identify methylation of the DKK1 promoter as a mechanism underlying the absence of DKK1 expression in advanced stage MM. These data strongly suggest that epigenetic silencing of DKK1 unleashes Wnt signaling in a subset of advanced myelomas, promoting disease progression.

## Supporting Information

Figure S1 **Bisulfite genomic sequencing of the** ***DKK1*** **promoter in MM cell line.** Representative pictures of immunocytochemical staining of multiple myeloma cell lines with goat polyclonal anti-DKK1 antibody (magnification: 400×). Prostate cancer cell line (PC-3) was used as positive control for the DKK1 staining.
(TIF)

Figure S2 **Bisulfite genomic sequencing of the** ***DKK1*** **promoter in MM bone marrow samples.** Bisulfite sequencing analysis was performed on DNA isolated from total bone marrow samples of twelve MM patients (P1–P12). For individual patient 5 clones of the *DKK1* promoter region are presented. Open circles indicate unmethylated CpG sites; closed circles represent methylated CpG sites.
(TIF)

Table S1 **Expression of Wnt family members in 345 MM patients from total therapy 2 (TT2) patients set.**
(DOC)
