# Supplementary figures and images for: Transcriptional Silencing of the Wnt-Antagonist DKK1 by Promoter Methylation Is Associated with Enhanced Wnt Signaling in Advanced Multiple Myeloma
Source: PLoS One. 2012 Feb 17;7(2):e30359. doi: 10.1371/journal.pone.0030359 (PMC3281831; doi:10.1371/journal.pone.0030359)

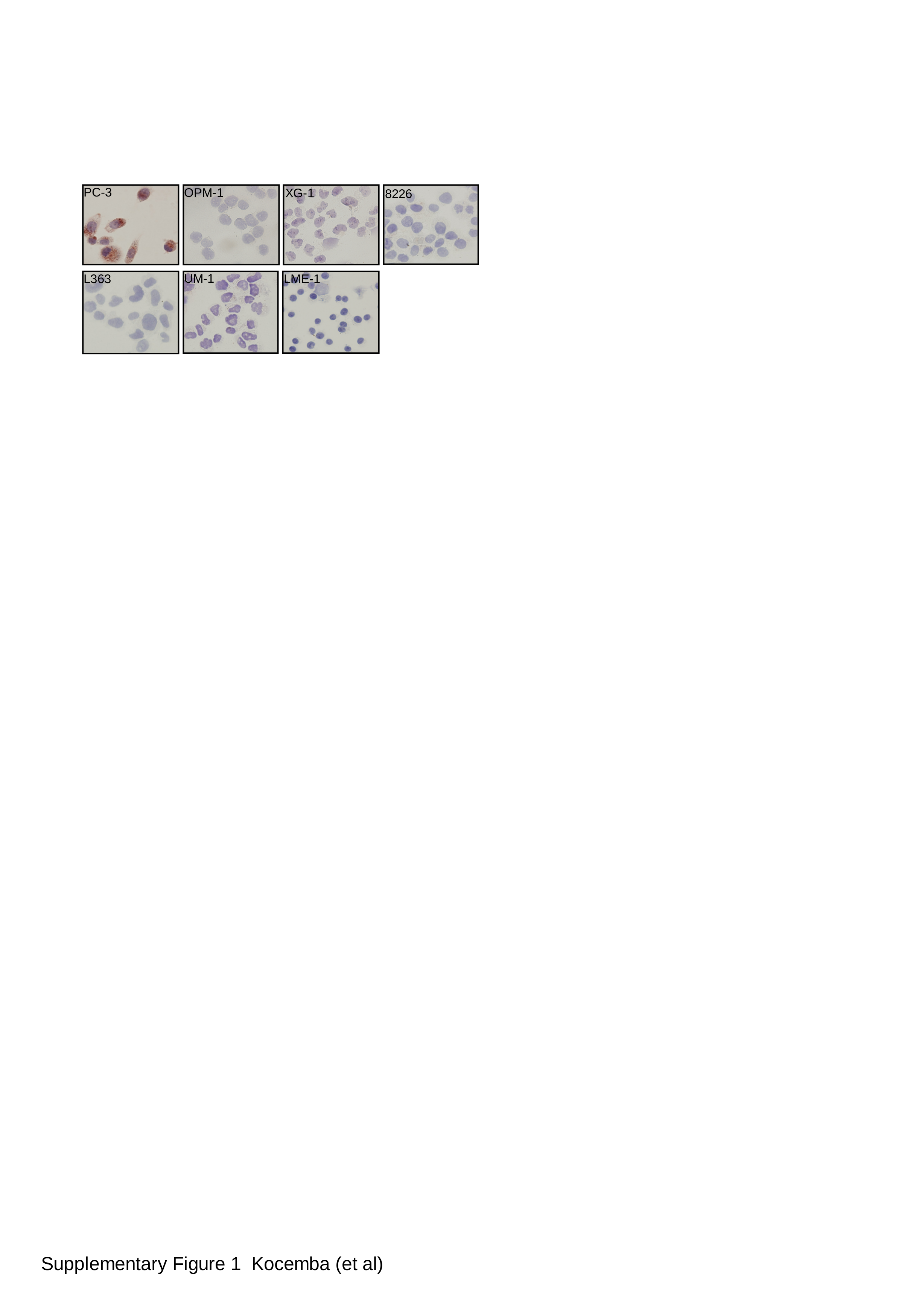

Supplement: Figure S1 — Bisulfite genomic sequencing of the DKK1 promoter in MM cell line. Representative pictures of immunocytochemical staining of multiple myeloma cell lines with goat polyclonal anti-DKK1 antibody (magnification: 400×). Prostate cancer cell line (PC-3) was used as positive control for the DKK1 staining. (TIF) [file pone.0030359.s001.tif]

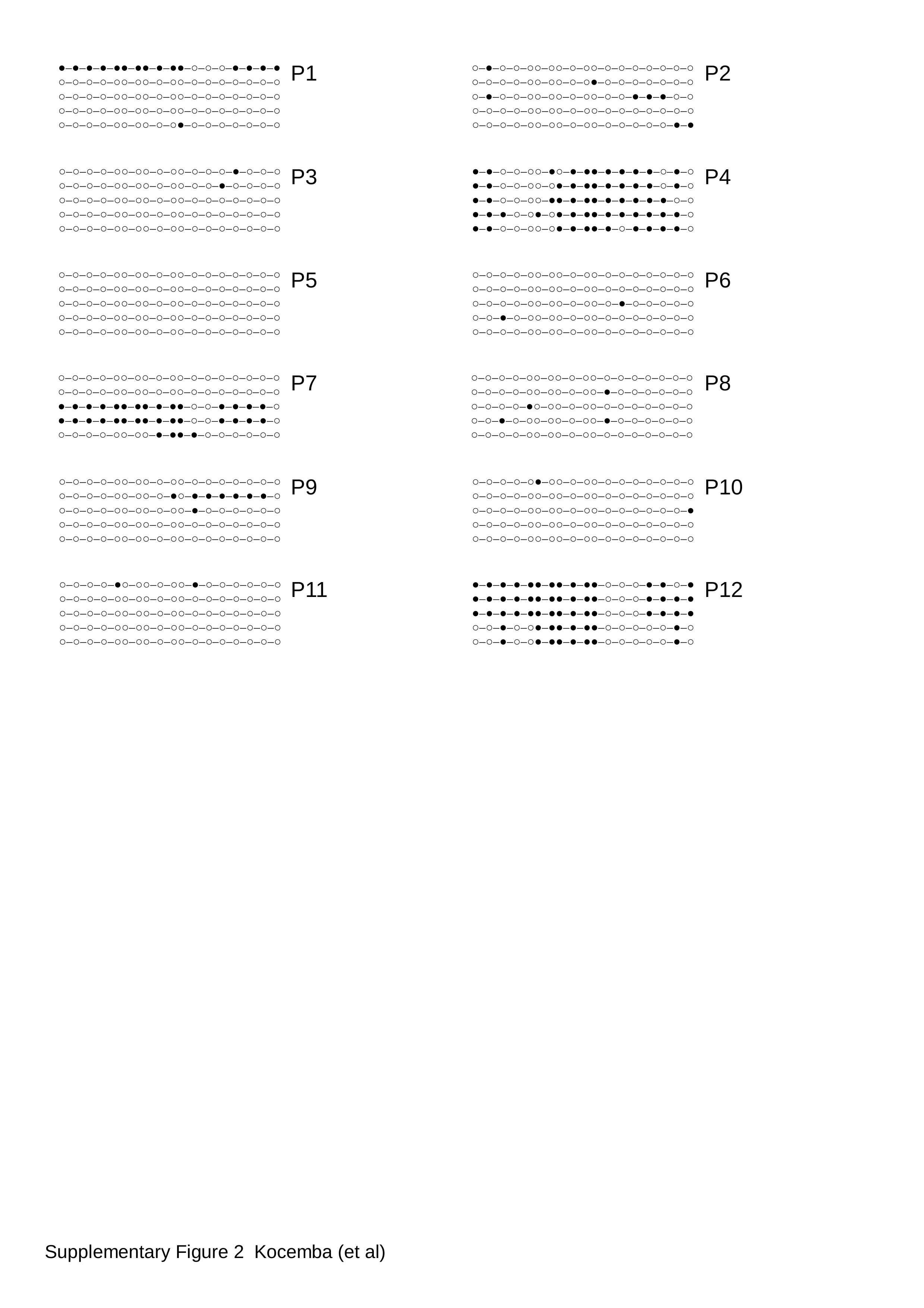

Supplement: Figure S2 — Bisulfite genomic sequencing of the DKK1 promoter in MM bone marrow samples. Bisulfite sequencing analysis was performed on DNA isolated from total bone marrow samples of twelve MM patients (P1–P12). For individual patient 5 clones of the DKK1 promoter region are presented. Open circles indicate unmethylated CpG sites; closed circles represent methylated CpG sites. (TIF) [file pone.0030359.s002.tif]
