# Supplementary material for: Transcriptional Silencing of the Wnt-Antagonist DKK1 by Promoter Methylation Is Associated with Enhanced Wnt Signaling in Advanced Multiple Myeloma
Source: PLoS One. 2012 Feb 17;7(2):e30359. doi: 10.1371/journal.pone.0030359 (PMC3281831; doi:10.1371/journal.pone.0030359)
Supplement: Table S1 — Expression of Wnt family members in 345 MM patients from total therapy 2 (TT2) patients set. (DOC) [file pone.0030359.s003.doc]

| **MM** **PATIENTS** | LRP6 | FZD1 | FZD2 | FZD3 | FZD4 | FZD6 | FZD7 | FZD8 | WNT2 | WNT2B | WNT3 | WNT4 | WNT5A | WNT5B | WNT6 | WNT7A | WNT8A | WNT10A | WNT11 | WNT16 |
| --- | --- | --- | --- | --- | --- | --- | --- | --- | --- | --- | --- | --- | --- | --- | --- | --- | --- | --- | --- | --- |
| MMJS50986 |  |  |  |  |  |  |  |  |  |  |  |  |  |  |  |  |  |  |  |  |
| MMJS50987 |  |  |  |  |  |  |  |  |  |  |  |  |  |  |  |  |  |  |  |  |
| MMJS50988 |  |  |  |  |  |  |  |  |  |  |  |  |  |  |  |  |  |  |  |  |
| MMJS50989 |  |  |  |  |  |  |  |  |  |  |  |  |  |  |  |  |  |  |  |  |
| MMJS50990 |  |  |  |  |  |  |  |  |  |  |  |  |  |  |  |  |  |  |  |  |
| MMJS50991 |  |  |  |  |  |  |  |  |  |  |  |  |  |  |  |  |  |  |  |  |
| MMJS50992 |  |  |  |  |  |  |  |  |  |  |  |  |  |  |  |  |  |  |  |  |
| MMJS50993 |  |  |  |  |  |  |  |  |  |  |  |  |  |  |  |  |  |  |  |  |
| MMJS50994 |  |  |  |  |  |  |  |  |  |  |  |  |  |  |  |  |  |  |  |  |
| MMJS50995 |  |  |  |  |  |  |  |  |  |  |  |  |  |  |  |  |  |  |  |  |
| MMJS50996 |  |  |  |  |  |  |  |  |  |  |  |  |  |  |  |  |  |  |  |  |
| MMJS50997 |  |  |  |  |  |  |  |  |  |  |  |  |  |  |  |  |  |  |  |  |
| MMJS50998 |  |  |  |  |  |  |  |  |  |  |  |  |  |  |  |  |  |  |  |  |
| MMJS50999 |  |  |  |  |  |  |  |  |  |  |  |  |  |  |  |  |  |  |  |  |
| MMJS51000 |  |  |  |  |  |  |  |  |  |  |  |  |  |  |  |  |  |  |  |  |
| MMJS51001 |  |  |  |  |  |  |  |  |  |  |  |  |  |  |  |  |  |  |  |  |
| MMJS51002 |  |  |  |  |  |  |  |  |  |  |  |  |  |  |  |  |  |  |  |  |
| MMJS51003 |  |  |  |  |  |  |  |  |  |  |  |  |  |  |  |  |  |  |  |  |
| MMJS51004 |  |  |  |  |  |  |  |  |  |  |  |  |  |  |  |  |  |  |  |  |
| MMJS51005 |  |  |  |  |  |  |  |  |  |  |  |  |  |  |  |  |  |  |  |  |
| MMJS51006 |  |  |  |  |  |  |  |  |  |  |  |  |  |  |  |  |  |  |  |  |
| MMJS51007 |  |  |  |  |  |  |  |  |  |  |  |  |  |  |  |  |  |  |  |  |
| MMJS51008 |  |  |  |  |  |  |  |  |  |  |  |  |  |  |  |  |  |  |  |  |
| MMJS51009 |  |  |  |  |  |  |  |  |  |  |  |  |  |  |  |  |  |  |  |  |
| MMJS51010 |  |  |  |  |  |  |  |  |  |  |  |  |  |  |  |  |  |  |  |  |
| MMJS51011 |  |  |  |  |  |  |  |  |  |  |  |  |  |  |  |  |  |  |  |  |
| MMJS51012 |  |  |  |  |  |  |  |  |  |  |  |  |  |  |  |  |  |  |  |  |
| MMJS51013 |  |  |  |  |  |  |  |  |  |  |  |  |  |  |  |  |  |  |  |  |
| MMJS51014 |  |  |  |  |  |  |  |  |  |  |  |  |  |  |  |  |  |  |  |  |
| MMJS51015 |  |  |  |  |  |  |  |  |  |  |  |  |  |  |  |  |  |  |  |  |
| MMJS51016 |  |  |  |  |  |  |  |  |  |  |  |  |  |  |  |  |  |  |  |  |
| MMJS51017 |  |  |  |  |  |  |  |  |  |  |  |  |  |  |  |  |  |  |  |  |
| MMJS51018 |  |  |  |  |  |  |  |  |  |  |  |  |  |  |  |  |  |  |  |  |
| MMJS51019 |  |  |  |  |  |  |  |  |  |  |  |  |  |  |  |  |  |  |  |  |
| MMJS51020 |  |  |  |  |  |  |  |  |  |  |  |  |  |  |  |  |  |  |  |  |
| MMJS51021 |  |  |  |  |  |  |  |  |  |  |  |  |  |  |  |  |  |  |  |  |
| MMJS51022 |  |  |  |  |  |  |  |  |  |  |  |  |  |  |  |  |  |  |  |  |
| MMJS51023 |  |  |  |  |  |  |  |  |  |  |  |  |  |  |  |  |  |  |  |  |
| MMJS51024 |  |  |  |  |  |  |  |  |  |  |  |  |  |  |  |  |  |  |  |  |
| MMJS51025 |  |  |  |  |  |  |  |  |  |  |  |  |  |  |  |  |  |  |  |  |
| MMJS51026 |  |  |  |  |  |  |  |  |  |  |  |  |  |  |  |  |  |  |  |  |
| MMJS51027 |  |  |  |  |  |  |  |  |  |  |  |  |  |  |  |  |  |  |  |  |
| MMJS51028 |  |  |  |  |  |  |  |  |  |  |  |  |  |  |  |  |  |  |  |  |
| MMJS51029 |  |  |  |  |  |  |  |  |  |  |  |  |  |  |  |  |  |  |  |  |
| MMJS51030 |  |  |  |  |  |  |  |  |  |  |  |  |  |  |  |  |  |  |  |  |
| MMJS51031 |  |  |  |  |  |  |  |  |  |  |  |  |  |  |  |  |  |  |  |  |
| MMJS51032 |  |  |  |  |  |  |  |  |  |  |  |  |  |  |  |  |  |  |  |  |
| MMJS51033 |  |  |  |  |  |  |  |  |  |  |  |  |  |  |  |  |  |  |  |  |
| MMJS51034 |  |  |  |  |  |  |  |  |  |  |  |  |  |  |  |  |  |  |  |  |
| MMJS51035 |  |  |  |  |  |  |  |  |  |  |  |  |  |  |  |  |  |  |  |  |
| MMJS51036 |  |  |  |  |  |  |  |  |  |  |  |  |  |  |  |  |  |  |  |  |
| MMJS51037 |  |  |  |  |  |  |  |  |  |  |  |  |  |  |  |  |  |  |  |  |
| MMJS51038 |  |  |  |  |  |  |  |  |  |  |  |  |  |  |  |  |  |  |  |  |
| MMJS51039 |  |  |  |  |  |  |  |  |  |  |  |  |  |  |  |  |  |  |  |  |
| MMJS51040 |  |  |  |  |  |  |  |  |  |  |  |  |  |  |  |  |  |  |  |  |
| MMJS51041 |  |  |  |  |  |  |  |  |  |  |  |  |  |  |  |  |  |  |  |  |
| MMJS51042 |  |  |  |  |  |  |  |  |  |  |  |  |  |  |  |  |  |  |  |  |
| MMJS51043 |  |  |  |  |  |  |  |  |  |  |  |  |  |  |  |  |  |  |  |  |
| MMJS51044 |  |  |  |  |  |  |  |  |  |  |  |  |  |  |  |  |  |  |  |  |
| MMJS51045 |  |  |  |  |  |  |  |  |  |  |  |  |  |  |  |  |  |  |  |  |
| MMJS51046 |  |  |  |  |  |  |  |  |  |  |  |  |  |  |  |  |  |  |  |  |
| MMJS51047 |  |  |  |  |  |  |  |  |  |  |  |  |  |  |  |  |  |  |  |  |
| MMJS51048 |  |  |  |  |  |  |  |  |  |  |  |  |  |  |  |  |  |  |  |  |
| MMJS51049 |  |  |  |  |  |  |  |  |  |  |  |  |  |  |  |  |  |  |  |  |
| MMJS51050 |  |  |  |  |  |  |  |  |  |  |  |  |  |  |  |  |  |  |  |  |
| MMJS51051 |  |  |  |  |  |  |  |  |  |  |  |  |  |  |  |  |  |  |  |  |
| MMJS51052 |  |  |  |  |  |  |  |  |  |  |  |  |  |  |  |  |  |  |  |  |
| MMJS51053 |  |  |  |  |  |  |  |  |  |  |  |  |  |  |  |  |  |  |  |  |
| MMJS51054 |  |  |  |  |  |  |  |  |  |  |  |  |  |  |  |  |  |  |  |  |
| MMJS51055 |  |  |  |  |  |  |  |  |  |  |  |  |  |  |  |  |  |  |  |  |
| MMJS51056 |  |  |  |  |  |  |  |  |  |  |  |  |  |  |  |  |  |  |  |  |
| MMJS51057 |  |  |  |  |  |  |  |  |  |  |  |  |  |  |  |  |  |  |  |  |
| MMJS51058 |  |  |  |  |  |  |  |  |  |  |  |  |  |  |  |  |  |  |  |  |
| MMJS51059 |  |  |  |  |  |  |  |  |  |  |  |  |  |  |  |  |  |  |  |  |
| MMJS51060 |  |  |  |  |  |  |  |  |  |  |  |  |  |  |  |  |  |  |  |  |
| MMJS51061 |  |  |  |  |  |  |  |  |  |  |  |  |  |  |  |  |  |  |  |  |
| MMJS51062 |  |  |  |  |  |  |  |  |  |  |  |  |  |  |  |  |  |  |  |  |
| MMJS51063 |  |  |  |  |  |  |  |  |  |  |  |  |  |  |  |  |  |  |  |  |
| MMJS51064 |  |  |  |  |  |  |  |  |  |  |  |  |  |  |  |  |  |  |  |  |
| MMJS51065 |  |  |  |  |  |  |  |  |  |  |  |  |  |  |  |  |  |  |  |  |
| MMJS51066 |  |  |  |  |  |  |  |  |  |  |  |  |  |  |  |  |  |  |  |  |
| MMJS51067 |  |  |  |  |  |  |  |  |  |  |  |  |  |  |  |  |  |  |  |  |
| MMJS51068 |  |  |  |  |  |  |  |  |  |  |  |  |  |  |  |  |  |  |  |  |
| MMJS51069 |  |  |  |  |  |  |  |  |  |  |  |  |  |  |  |  |  |  |  |  |
| MMJS51070 |  |  |  |  |  |  |  |  |  |  |  |  |  |  |  |  |  |  |  |  |
| MMJS51071 |  |  |  |  |  |  |  |  |  |  |  |  |  |  |  |  |  |  |  |  |
| MMJS51072 |  |  |  |  |  |  |  |  |  |  |  |  |  |  |  |  |  |  |  |  |
| MMJS51073 |  |  |  |  |  |  |  |  |  |  |  |  |  |  |  |  |  |  |  |  |
| MMJS51074 |  |  |  |  |  |  |  |  |  |  |  |  |  |  |  |  |  |  |  |  |
| MMJS51075 |  |  |  |  |  |  |  |  |  |  |  |  |  |  |  |  |  |  |  |  |
| MMJS51076 |  |  |  |  |  |  |  |  |  |  |  |  |  |  |  |  |  |  |  |  |
| MMJS51077 |  |  |  |  |  |  |  |  |  |  |  |  |  |  |  |  |  |  |  |  |
| MMJS51078 |  |  |  |  |  |  |  |  |  |  |  |  |  |  |  |  |  |  |  |  |
| MMJS51079 |  |  |  |  |  |  |  |  |  |  |  |  |  |  |  |  |  |  |  |  |
| MMJS51080 |  |  |  |  |  |  |  |  |  |  |  |  |  |  |  |  |  |  |  |  |
| MMJS51081 |  |  |  |  |  |  |  |  |  |  |  |  |  |  |  |  |  |  |  |  |
| MMJS51082 |  |  |  |  |  |  |  |  |  |  |  |  |  |  |  |  |  |  |  |  |
| MMJS51083 |  |  |  |  |  |  |  |  |  |  |  |  |  |  |  |  |  |  |  |  |
| MMJS51084 |  |  |  |  |  |  |  |  |  |  |  |  |  |  |  |  |  |  |  |  |
| MMJS51085 |  |  |  |  |  |  |  |  |  |  |  |  |  |  |  |  |  |  |  |  |
| MMJS51086 |  |  |  |  |  |  |  |  |  |  |  |  |  |  |  |  |  |  |  |  |
| MMJS51087 |  |  |  |  |  |  |  |  |  |  |  |  |  |  |  |  |  |  |  |  |
| MMJS51088 |  |  |  |  |  |  |  |  |  |  |  |  |  |  |  |  |  |  |  |  |
| MMJS51089 |  |  |  |  |  |  |  |  |  |  |  |  |  |  |  |  |  |  |  |  |
| MMJS51090 |  |  |  |  |  |  |  |  |  |  |  |  |  |  |  |  |  |  |  |  |
| MMJS51091 |  |  |  |  |  |  |  |  |  |  |  |  |  |  |  |  |  |  |  |  |
| MMJS51092 |  |  |  |  |  |  |  |  |  |  |  |  |  |  |  |  |  |  |  |  |
| MMJS51093 |  |  |  |  |  |  |  |  |  |  |  |  |  |  |  |  |  |  |  |  |
| MMJS51094 |  |  |  |  |  |  |  |  |  |  |  |  |  |  |  |  |  |  |  |  |
| MMJS51095 |  |  |  |  |  |  |  |  |  |  |  |  |  |  |  |  |  |  |  |  |
| MMJS51096 |  |  |  |  |  |  |  |  |  |  |  |  |  |  |  |  |  |  |  |  |
| MMJS51097 |  |  |  |  |  |  |  |  |  |  |  |  |  |  |  |  |  |  |  |  |
| MMJS51098 |  |  |  |  |  |  |  |  |  |  |  |  |  |  |  |  |  |  |  |  |
| MMJS51099 |  |  |  |  |  |  |  |  |  |  |  |  |  |  |  |  |  |  |  |  |
| MMJS51100 |  |  |  |  |  |  |  |  |  |  |  |  |  |  |  |  |  |  |  |  |
| MMJS51101 |  |  |  |  |  |  |  |  |  |  |  |  |  |  |  |  |  |  |  |  |
| MMJS51102 |  |  |  |  |  |  |  |  |  |  |  |  |  |  |  |  |  |  |  |  |
| MMJS51103 |  |  |  |  |  |  |  |  |  |  |  |  |  |  |  |  |  |  |  |  |
| MMJS51104 |  |  |  |  |  |  |  |  |  |  |  |  |  |  |  |  |  |  |  |  |
| MMJS51105 |  |  |  |  |  |  |  |  |  |  |  |  |  |  |  |  |  |  |  |  |
| MMJS51106 |  |  |  |  |  |  |  |  |  |  |  |  |  |  |  |  |  |  |  |  |
| MMJS51107 |  |  |  |  |  |  |  |  |  |  |  |  |  |  |  |  |  |  |  |  |
| MMJS51108 |  |  |  |  |  |  |  |  |  |  |  |  |  |  |  |  |  |  |  |  |
| MMJS51109 |  |  |  |  |  |  |  |  |  |  |  |  |  |  |  |  |  |  |  |  |
| MMJS51110 |  |  |  |  |  |  |  |  |  |  |  |  |  |  |  |  |  |  |  |  |
| MMJS51111 |  |  |  |  |  |  |  |  |  |  |  |  |  |  |  |  |  |  |  |  |
| MMJS51112 |  |  |  |  |  |  |  |  |  |  |  |  |  |  |  |  |  |  |  |  |
| MMJS51113 |  |  |  |  |  |  |  |  |  |  |  |  |  |  |  |  |  |  |  |  |
| MMJS51114 |  |  |  |  |  |  |  |  |  |  |  |  |  |  |  |  |  |  |  |  |
| MMJS51115 |  |  |  |  |  |  |  |  |  |  |  |  |  |  |  |  |  |  |  |  |
| MMJS51116 |  |  |  |  |  |  |  |  |  |  |  |  |  |  |  |  |  |  |  |  |
| MMJS51117 |  |  |  |  |  |  |  |  |  |  |  |  |  |  |  |  |  |  |  |  |
| MMJS51118 |  |  |  |  |  |  |  |  |  |  |  |  |  |  |  |  |  |  |  |  |
| MMJS51119 |  |  |  |  |  |  |  |  |  |  |  |  |  |  |  |  |  |  |  |  |
| MMJS51120 |  |  |  |  |  |  |  |  |  |  |  |  |  |  |  |  |  |  |  |  |
| MMJS51121 |  |  |  |  |  |  |  |  |  |  |  |  |  |  |  |  |  |  |  |  |
| MMJS51122 |  |  |  |  |  |  |  |  |  |  |  |  |  |  |  |  |  |  |  |  |
| MMJS51123 |  |  |  |  |  |  |  |  |  |  |  |  |  |  |  |  |  |  |  |  |
| MMJS51124 |  |  |  |  |  |  |  |  |  |  |  |  |  |  |  |  |  |  |  |  |
| MMJS51125 |  |  |  |  |  |  |  |  |  |  |  |  |  |  |  |  |  |  |  |  |
| MMJS51126 |  |  |  |  |  |  |  |  |  |  |  |  |  |  |  |  |  |  |  |  |
| MMJS51127 |  |  |  |  |  |  |  |  |  |  |  |  |  |  |  |  |  |  |  |  |
| MMJS51128 |  |  |  |  |  |  |  |  |  |  |  |  |  |  |  |  |  |  |  |  |
| MMJS51129 |  |  |  |  |  |  |  |  |  |  |  |  |  |  |  |  |  |  |  |  |
| MMJS51130 |  |  |  |  |  |  |  |  |  |  |  |  |  |  |  |  |  |  |  |  |
| MMJS51131 |  |  |  |  |  |  |  |  |  |  |  |  |  |  |  |  |  |  |  |  |
| MMJS51132 |  |  |  |  |  |  |  |  |  |  |  |  |  |  |  |  |  |  |  |  |
| MMJS51133 |  |  |  |  |  |  |  |  |  |  |  |  |  |  |  |  |  |  |  |  |
| MMJS51134 |  |  |  |  |  |  |  |  |  |  |  |  |  |  |  |  |  |  |  |  |
| MMJS51135 |  |  |  |  |  |  |  |  |  |  |  |  |  |  |  |  |  |  |  |  |
| MMJS51136 |  |  |  |  |  |  |  |  |  |  |  |  |  |  |  |  |  |  |  |  |
| MMJS51137 |  |  |  |  |  |  |  |  |  |  |  |  |  |  |  |  |  |  |  |  |
| MMJS51138 |  |  |  |  |  |  |  |  |  |  |  |  |  |  |  |  |  |  |  |  |
| MMJS51139 |  |  |  |  |  |  |  |  |  |  |  |  |  |  |  |  |  |  |  |  |
| MMJS51140 |  |  |  |  |  |  |  |  |  |  |  |  |  |  |  |  |  |  |  |  |
| MMJS51141 |  |  |  |  |  |  |  |  |  |  |  |  |  |  |  |  |  |  |  |  |
| MMJS51142 |  |  |  |  |  |  |  |  |  |  |  |  |  |  |  |  |  |  |  |  |
| MMJS51143 |  |  |  |  |  |  |  |  |  |  |  |  |  |  |  |  |  |  |  |  |
| MMJS51144 |  |  |  |  |  |  |  |  |  |  |  |  |  |  |  |  |  |  |  |  |
| MMJS51145 |  |  |  |  |  |  |  |  |  |  |  |  |  |  |  |  |  |  |  |  |
| MMJS51146 |  |  |  |  |  |  |  |  |  |  |  |  |  |  |  |  |  |  |  |  |
| MMJS51147 |  |  |  |  |  |  |  |  |  |  |  |  |  |  |  |  |  |  |  |  |
| MMJS51148 |  |  |  |  |  |  |  |  |  |  |  |  |  |  |  |  |  |  |  |  |
| MMJS51149 |  |  |  |  |  |  |  |  |  |  |  |  |  |  |  |  |  |  |  |  |
| MMJS51150 |  |  |  |  |  |  |  |  |  |  |  |  |  |  |  |  |  |  |  |  |
| MMJS51151 |  |  |  |  |  |  |  |  |  |  |  |  |  |  |  |  |  |  |  |  |
| MMJS51152 |  |  |  |  |  |  |  |  |  |  |  |  |  |  |  |  |  |  |  |  |
| MMJS51153 |  |  |  |  |  |  |  |  |  |  |  |  |  |  |  |  |  |  |  |  |
| MMJS51154 |  |  |  |  |  |  |  |  |  |  |  |  |  |  |  |  |  |  |  |  |
| MMJS51155 |  |  |  |  |  |  |  |  |  |  |  |  |  |  |  |  |  |  |  |  |
| MMJS51156 |  |  |  |  |  |  |  |  |  |  |  |  |  |  |  |  |  |  |  |  |
| MMJS51157 |  |  |  |  |  |  |  |  |  |  |  |  |  |  |  |  |  |  |  |  |
| MMJS51158 |  |  |  |  |  |  |  |  |  |  |  |  |  |  |  |  |  |  |  |  |
| MMJS51159 |  |  |  |  |  |  |  |  |  |  |  |  |  |  |  |  |  |  |  |  |
| MMJS51160 |  |  |  |  |  |  |  |  |  |  |  |  |  |  |  |  |  |  |  |  |
| MMJS51161 |  |  |  |  |  |  |  |  |  |  |  |  |  |  |  |  |  |  |  |  |
| MMJS51162 |  |  |  |  |  |  |  |  |  |  |  |  |  |  |  |  |  |  |  |  |
| MMJS51163 |  |  |  |  |  |  |  |  |  |  |  |  |  |  |  |  |  |  |  |  |
| MMJS51164 |  |  |  |  |  |  |  |  |  |  |  |  |  |  |  |  |  |  |  |  |
| MMJS51165 |  |  |  |  |  |  |  |  |  |  |  |  |  |  |  |  |  |  |  |  |
| MMJS51166 |  |  |  |  |  |  |  |  |  |  |  |  |  |  |  |  |  |  |  |  |
| MMJS51167 |  |  |  |  |  |  |  |  |  |  |  |  |  |  |  |  |  |  |  |  |
| MMJS51168 |  |  |  |  |  |  |  |  |  |  |  |  |  |  |  |  |  |  |  |  |
| MMJS51169 |  |  |  |  |  |  |  |  |  |  |  |  |  |  |  |  |  |  |  |  |
| MMJS51170 |  |  |  |  |  |  |  |  |  |  |  |  |  |  |  |  |  |  |  |  |
| MMJS51171 |  |  |  |  |  |  |  |  |  |  |  |  |  |  |  |  |  |  |  |  |
| MMJS51172 |  |  |  |  |  |  |  |  |  |  |  |  |  |  |  |  |  |  |  |  |
| MMJS51173 |  |  |  |  |  |  |  |  |  |  |  |  |  |  |  |  |  |  |  |  |
| MMJS51174 |  |  |  |  |  |  |  |  |  |  |  |  |  |  |  |  |  |  |  |  |
| MMJS51175 |  |  |  |  |  |  |  |  |  |  |  |  |  |  |  |  |  |  |  |  |
| MMJS51176 |  |  |  |  |  |  |  |  |  |  |  |  |  |  |  |  |  |  |  |  |
| MMJS51177 |  |  |  |  |  |  |  |  |  |  |  |  |  |  |  |  |  |  |  |  |
| MMJS51178 |  |  |  |  |  |  |  |  |  |  |  |  |  |  |  |  |  |  |  |  |
| MMJS51179 |  |  |  |  |  |  |  |  |  |  |  |  |  |  |  |  |  |  |  |  |
| MMJS51180 |  |  |  |  |  |  |  |  |  |  |  |  |  |  |  |  |  |  |  |  |
| MMJS51181 |  |  |  |  |  |  |  |  |  |  |  |  |  |  |  |  |  |  |  |  |
| MMJS51182 |  |  |  |  |  |  |  |  |  |  |  |  |  |  |  |  |  |  |  |  |
| MMJS51183 |  |  |  |  |  |  |  |  |  |  |  |  |  |  |  |  |  |  |  |  |
| MMJS51184 |  |  |  |  |  |  |  |  |  |  |  |  |  |  |  |  |  |  |  |  |
| MMJS51185 |  |  |  |  |  |  |  |  |  |  |  |  |  |  |  |  |  |  |  |  |
| **MMJS51186** |  |  |  |  |  |  |  |  |  |  |  |  |  |  |  |  |  |  |  |  |
| **MMJS51187** |  |  |  |  |  |  |  |  |  |  |  |  |  |  |  |  |  |  |  |  |
| **MMJS51188** |  |  |  |  |  |  |  |  |  |  |  |  |  |  |  |  |  |  |  |  |
| **MMJS51189** |  |  |  |  |  |  |  |  |  |  |  |  |  |  |  |  |  |  |  |  |
| **MMJS51190** |  |  |  |  |  |  |  |  |  |  |  |  |  |  |  |  |  |  |  |  |
| **MMJS51191** |  |  |  |  |  |  |  |  |  |  |  |  |  |  |  |  |  |  |  |  |

| **MM PATIENTS** | LRP6 | FZD1 | FZD2 | FZD3 | FZD4 | FZD6 | FZD7 | FZD8 | WNT2 | WNT2B | WNT3 | WNT4 | WNT5A | WNT5B | WNT6 | WNT7A | WNT8A | WNT10A | WNT11 | WNT16 |
| --- | --- | --- | --- | --- | --- | --- | --- | --- | --- | --- | --- | --- | --- | --- | --- | --- | --- | --- | --- | --- |
| MMJS51192 |  |  |  |  |  |  |  |  |  |  |  |  |  |  |  |  |  |  |  |  |
| MMJS51193 |  |  |  |  |  |  |  |  |  |  |  |  |  |  |  |  |  |  |  |  |
| MMJS51194 |  |  |  |  |  |  |  |  |  |  |  |  |  |  |  |  |  |  |  |  |
| MMJS51195 |  |  |  |  |  |  |  |  |  |  |  |  |  |  |  |  |  |  |  |  |
| MMJS51196 |  |  |  |  |  |  |  |  |  |  |  |  |  |  |  |  |  |  |  |  |
| MMJS51197 |  |  |  |  |  |  |  |  |  |  |  |  |  |  |  |  |  |  |  |  |
| MMJS51198 |  |  |  |  |  |  |  |  |  |  |  |  |  |  |  |  |  |  |  |  |
| MMJS51199 |  |  |  |  |  |  |  |  |  |  |  |  |  |  |  |  |  |  |  |  |
| MMJS51200 |  |  |  |  |  |  |  |  |  |  |  |  |  |  |  |  |  |  |  |  |
| MMJS51201 |  |  |  |  |  |  |  |  |  |  |  |  |  |  |  |  |  |  |  |  |
| MMJS51202 |  |  |  |  |  |  |  |  |  |  |  |  |  |  |  |  |  |  |  |  |
| MMJS51203 |  |  |  |  |  |  |  |  |  |  |  |  |  |  |  |  |  |  |  |  |
| MMJS51204 |  |  |  |  |  |  |  |  |  |  |  |  |  |  |  |  |  |  |  |  |
| MMJS51205 |  |  |  |  |  |  |  |  |  |  |  |  |  |  |  |  |  |  |  |  |
| MMJS51206 |  |  |  |  |  |  |  |  |  |  |  |  |  |  |  |  |  |  |  |  |
| MMJS51207 |  |  |  |  |  |  |  |  |  |  |  |  |  |  |  |  |  |  |  |  |
| MMJS51208 |  |  |  |  |  |  |  |  |  |  |  |  |  |  |  |  |  |  |  |  |
| MMJS51209 |  |  |  |  |  |  |  |  |  |  |  |  |  |  |  |  |  |  |  |  |
| MMJS51210 |  |  |  |  |  |  |  |  |  |  |  |  |  |  |  |  |  |  |  |  |
| MMJS51211 |  |  |  |  |  |  |  |  |  |  |  |  |  |  |  |  |  |  |  |  |
| MMJS51212 |  |  |  |  |  |  |  |  |  |  |  |  |  |  |  |  |  |  |  |  |
| MMJS51213 |  |  |  |  |  |  |  |  |  |  |  |  |  |  |  |  |  |  |  |  |
| MMJS51214 |  |  |  |  |  |  |  |  |  |  |  |  |  |  |  |  |  |  |  |  |
| MMJS51215 |  |  |  |  |  |  |  |  |  |  |  |  |  |  |  |  |  |  |  |  |
| MMJS51216 |  |  |  |  |  |  |  |  |  |  |  |  |  |  |  |  |  |  |  |  |
| MMJS51217 |  |  |  |  |  |  |  |  |  |  |  |  |  |  |  |  |  |  |  |  |
| MMJS51218 |  |  |  |  |  |  |  |  |  |  |  |  |  |  |  |  |  |  |  |  |
| MMJS51219 |  |  |  |  |  |  |  |  |  |  |  |  |  |  |  |  |  |  |  |  |
| MMJS51220 |  |  |  |  |  |  |  |  |  |  |  |  |  |  |  |  |  |  |  |  |
| MMJS51221 |  |  |  |  |  |  |  |  |  |  |  |  |  |  |  |  |  |  |  |  |
| MMJS51222 |  |  |  |  |  |  |  |  |  |  |  |  |  |  |  |  |  |  |  |  |
| MMJS51223 |  |  |  |  |  |  |  |  |  |  |  |  |  |  |  |  |  |  |  |  |
| MMJS51224 |  |  |  |  |  |  |  |  |  |  |  |  |  |  |  |  |  |  |  |  |
| MMJS51225 |  |  |  |  |  |  |  |  |  |  |  |  |  |  |  |  |  |  |  |  |
| MMJS51226 |  |  |  |  |  |  |  |  |  |  |  |  |  |  |  |  |  |  |  |  |
| MMJS51227 |  |  |  |  |  |  |  |  |  |  |  |  |  |  |  |  |  |  |  |  |
| MMJS51228 |  |  |  |  |  |  |  |  |  |  |  |  |  |  |  |  |  |  |  |  |
| MMJS51229 |  |  |  |  |  |  |  |  |  |  |  |  |  |  |  |  |  |  |  |  |
| MMJS51230 |  |  |  |  |  |  |  |  |  |  |  |  |  |  |  |  |  |  |  |  |
| MMJS51231 |  |  |  |  |  |  |  |  |  |  |  |  |  |  |  |  |  |  |  |  |
| MMJS51232 |  |  |  |  |  |  |  |  |  |  |  |  |  |  |  |  |  |  |  |  |
| MMJS51233 |  |  |  |  |  |  |  |  |  |  |  |  |  |  |  |  |  |  |  |  |
| MMJS51234 |  |  |  |  |  |  |  |  |  |  |  |  |  |  |  |  |  |  |  |  |
| MMJS51235 |  |  |  |  |  |  |  |  |  |  |  |  |  |  |  |  |  |  |  |  |
| MMJS51236 |  |  |  |  |  |  |  |  |  |  |  |  |  |  |  |  |  |  |  |  |
| MMJS51237 |  |  |  |  |  |  |  |  |  |  |  |  |  |  |  |  |  |  |  |  |
| MMJS51238 |  |  |  |  |  |  |  |  |  |  |  |  |  |  |  |  |  |  |  |  |
| MMJS51239 |  |  |  |  |  |  |  |  |  |  |  |  |  |  |  |  |  |  |  |  |
| MMJS51240 |  |  |  |  |  |  |  |  |  |  |  |  |  |  |  |  |  |  |  |  |
| MMJS51241 |  |  |  |  |  |  |  |  |  |  |  |  |  |  |  |  |  |  |  |  |
| MMJS51242 |  |  |  |  |  |  |  |  |  |  |  |  |  |  |  |  |  |  |  |  |
| MMJS51243 |  |  |  |  |  |  |  |  |  |  |  |  |  |  |  |  |  |  |  |  |
| MMJS51244 |  |  |  |  |  |  |  |  |  |  |  |  |  |  |  |  |  |  |  |  |
| MMJS51245 |  |  |  |  |  |  |  |  |  |  |  |  |  |  |  |  |  |  |  |  |
| MMJS51246 |  |  |  |  |  |  |  |  |  |  |  |  |  |  |  |  |  |  |  |  |
| MMJS51247 |  |  |  |  |  |  |  |  |  |  |  |  |  |  |  |  |  |  |  |  |
| MMJS51248 |  |  |  |  |  |  |  |  |  |  |  |  |  |  |  |  |  |  |  |  |
| MMJS51249 |  |  |  |  |  |  |  |  |  |  |  |  |  |  |  |  |  |  |  |  |
| MMJS51250 |  |  |  |  |  |  |  |  |  |  |  |  |  |  |  |  |  |  |  |  |
| MMJS51251 |  |  |  |  |  |  |  |  |  |  |  |  |  |  |  |  |  |  |  |  |
| MMJS51252 |  |  |  |  |  |  |  |  |  |  |  |  |  |  |  |  |  |  |  |  |
| MMJS51253 |  |  |  |  |  |  |  |  |  |  |  |  |  |  |  |  |  |  |  |  |
| MMJS51254 |  |  |  |  |  |  |  |  |  |  |  |  |  |  |  |  |  |  |  |  |
| MMJS51255 |  |  |  |  |  |  |  |  |  |  |  |  |  |  |  |  |  |  |  |  |
| MMJS51256 |  |  |  |  |  |  |  |  |  |  |  |  |  |  |  |  |  |  |  |  |
| MMJS51257 |  |  |  |  |  |  |  |  |  |  |  |  |  |  |  |  |  |  |  |  |
| MMJS51258 |  |  |  |  |  |  |  |  |  |  |  |  |  |  |  |  |  |  |  |  |
| MMJS51259 |  |  |  |  |  |  |  |  |  |  |  |  |  |  |  |  |  |  |  |  |
| MMJS51260 |  |  |  |  |  |  |  |  |  |  |  |  |  |  |  |  |  |  |  |  |
| MMJS51261 |  |  |  |  |  |  |  |  |  |  |  |  |  |  |  |  |  |  |  |  |
| MMJS51262 |  |  |  |  |  |  |  |  |  |  |  |  |  |  |  |  |  |  |  |  |
| MMJS51263 |  |  |  |  |  |  |  |  |  |  |  |  |  |  |  |  |  |  |  |  |
| MMJS51264 |  |  |  |  |  |  |  |  |  |  |  |  |  |  |  |  |  |  |  |  |
| MMJS51265 |  |  |  |  |  |  |  |  |  |  |  |  |  |  |  |  |  |  |  |  |
| MMJS51266 |  |  |  |  |  |  |  |  |  |  |  |  |  |  |  |  |  |  |  |  |
| MMJS51267 |  |  |  |  |  |  |  |  |  |  |  |  |  |  |  |  |  |  |  |  |
| MMJS51268 |  |  |  |  |  |  |  |  |  |  |  |  |  |  |  |  |  |  |  |  |
| MMJS51269 |  |  |  |  |  |  |  |  |  |  |  |  |  |  |  |  |  |  |  |  |
| MMJS51270 |  |  |  |  |  |  |  |  |  |  |  |  |  |  |  |  |  |  |  |  |
| MMJS51271 |  |  |  |  |  |  |  |  |  |  |  |  |  |  |  |  |  |  |  |  |
| MMJS51272 |  |  |  |  |  |  |  |  |  |  |  |  |  |  |  |  |  |  |  |  |
| MMJS51273 |  |  |  |  |  |  |  |  |  |  |  |  |  |  |  |  |  |  |  |  |
| MMJS51274 |  |  |  |  |  |  |  |  |  |  |  |  |  |  |  |  |  |  |  |  |
| MMJS51275 |  |  |  |  |  |  |  |  |  |  |  |  |  |  |  |  |  |  |  |  |
| MMJS51276 |  |  |  |  |  |  |  |  |  |  |  |  |  |  |  |  |  |  |  |  |
| MMJS51277 |  |  |  |  |  |  |  |  |  |  |  |  |  |  |  |  |  |  |  |  |
| MMJS51278 |  |  |  |  |  |  |  |  |  |  |  |  |  |  |  |  |  |  |  |  |
| MMJS51279 |  |  |  |  |  |  |  |  |  |  |  |  |  |  |  |  |  |  |  |  |
| MMJS51280 |  |  |  |  |  |  |  |  |  |  |  |  |  |  |  |  |  |  |  |  |
| MMJS51281 |  |  |  |  |  |  |  |  |  |  |  |  |  |  |  |  |  |  |  |  |
| MMJS51282 |  |  |  |  |  |  |  |  |  |  |  |  |  |  |  |  |  |  |  |  |
| MMJS51285 |  |  |  |  |  |  |  |  |  |  |  |  |  |  |  |  |  |  |  |  |
| MMJS51288 |  |  |  |  |  |  |  |  |  |  |  |  |  |  |  |  |  |  |  |  |
| MMJS51289 |  |  |  |  |  |  |  |  |  |  |  |  |  |  |  |  |  |  |  |  |
| MMJS51292 |  |  |  |  |  |  |  |  |  |  |  |  |  |  |  |  |  |  |  |  |
| MMJS51293 |  |  |  |  |  |  |  |  |  |  |  |  |  |  |  |  |  |  |  |  |
| MMJS51294 |  |  |  |  |  |  |  |  |  |  |  |  |  |  |  |  |  |  |  |  |
| MMJS51295 |  |  |  |  |  |  |  |  |  |  |  |  |  |  |  |  |  |  |  |  |
| MMJS51296 |  |  |  |  |  |  |  |  |  |  |  |  |  |  |  |  |  |  |  |  |
| MMJS51297 |  |  |  |  |  |  |  |  |  |  |  |  |  |  |  |  |  |  |  |  |
| MMJS51298 |  |  |  |  |  |  |  |  |  |  |  |  |  |  |  |  |  |  |  |  |
| MMJS51299 |  |  |  |  |  |  |  |  |  |  |  |  |  |  |  |  |  |  |  |  |
| MMJS51300 |  |  |  |  |  |  |  |  |  |  |  |  |  |  |  |  |  |  |  |  |
| MMJS51301 |  |  |  |  |  |  |  |  |  |  |  |  |  |  |  |  |  |  |  |  |
| MMJS51302 |  |  |  |  |  |  |  |  |  |  |  |  |  |  |  |  |  |  |  |  |
| MMJS51303 |  |  |  |  |  |  |  |  |  |  |  |  |  |  |  |  |  |  |  |  |
| MMJS51304 |  |  |  |  |  |  |  |  |  |  |  |  |  |  |  |  |  |  |  |  |
| MMJS51305 |  |  |  |  |  |  |  |  |  |  |  |  |  |  |  |  |  |  |  |  |
| MMJS51306 |  |  |  |  |  |  |  |  |  |  |  |  |  |  |  |  |  |  |  |  |
| MMJS51307 |  |  |  |  |  |  |  |  |  |  |  |  |  |  |  |  |  |  |  |  |
| MMJS51308 |  |  |  |  |  |  |  |  |  |  |  |  |  |  |  |  |  |  |  |  |
| MMJS51309 |  |  |  |  |  |  |  |  |  |  |  |  |  |  |  |  |  |  |  |  |
| MMJS51310 |  |  |  |  |  |  |  |  |  |  |  |  |  |  |  |  |  |  |  |  |
| MMJS51311 |  |  |  |  |  |  |  |  |  |  |  |  |  |  |  |  |  |  |  |  |
| MMJS51312 |  |  |  |  |  |  |  |  |  |  |  |  |  |  |  |  |  |  |  |  |
| MMJS51313 |  |  |  |  |  |  |  |  |  |  |  |  |  |  |  |  |  |  |  |  |
| MMJS51314 |  |  |  |  |  |  |  |  |  |  |  |  |  |  |  |  |  |  |  |  |
| MMJS51315 |  |  |  |  |  |  |  |  |  |  |  |  |  |  |  |  |  |  |  |  |
| MMJS51316 |  |  |  |  |  |  |  |  |  |  |  |  |  |  |  |  |  |  |  |  |
| MMJS51317 |  |  |  |  |  |  |  |  |  |  |  |  |  |  |  |  |  |  |  |  |
| MMJS51318 |  |  |  |  |  |  |  |  |  |  |  |  |  |  |  |  |  |  |  |  |
| MMJS51319 |  |  |  |  |  |  |  |  |  |  |  |  |  |  |  |  |  |  |  |  |
| MMJS51320 |  |  |  |  |  |  |  |  |  |  |  |  |  |  |  |  |  |  |  |  |
| MMJS51321 |  |  |  |  |  |  |  |  |  |  |  |  |  |  |  |  |  |  |  |  |
| MMJS51322 |  |  |  |  |  |  |  |  |  |  |  |  |  |  |  |  |  |  |  |  |
| MMJS51323 |  |  |  |  |  |  |  |  |  |  |  |  |  |  |  |  |  |  |  |  |
| MMJS51324 |  |  |  |  |  |  |  |  |  |  |  |  |  |  |  |  |  |  |  |  |
| MMJS51325 |  |  |  |  |  |  |  |  |  |  |  |  |  |  |  |  |  |  |  |  |
| MMJS51326 |  |  |  |  |  |  |  |  |  |  |  |  |  |  |  |  |  |  |  |  |
| MMJS51327 |  |  |  |  |  |  |  |  |  |  |  |  |  |  |  |  |  |  |  |  |
| MMJS51328 |  |  |  |  |  |  |  |  |  |  |  |  |  |  |  |  |  |  |  |  |
| MMJS51329 |  |  |  |  |  |  |  |  |  |  |  |  |  |  |  |  |  |  |  |  |
| MMJS51330 |  |  |  |  |  |  |  |  |  |  |  |  |  |  |  |  |  |  |  |  |
| MMJS51331 |  |  |  |  |  |  |  |  |  |  |  |  |  |  |  |  |  |  |  |  |
| MMJS51332 |  |  |  |  |  |  |  |  |  |  |  |  |  |  |  |  |  |  |  |  |
| MMJS51333 |  |  |  |  |  |  |  |  |  |  |  |  |  |  |  |  |  |  |  |  |
| MMJS51334 |  |  |  |  |  |  |  |  |  |  |  |  |  |  |  |  |  |  |  |  |
| MMJS51335 |  |  |  |  |  |  |  |  |  |  |  |  |  |  |  |  |  |  |  |  |
| MMJS51336 |  |  |  |  |  |  |  |  |  |  |  |  |  |  |  |  |  |  |  |  |

Legend:

transcript is not detected (Absent)

transcript is marginally detected (Marginal)

transcript is detected (Present)
